# Supplementary material for: Identification and Total Synthesis of Two Previously Unreported Odd-Chain Bis-Methylene-Interrupted Fatty Acids with a Terminal Olefin that Activate Protein Phosphatase, Mg2+/Mn2+-Dependent 1A (PPM1A) in Ovaries of the Limpet Cellana toreuma
Source: Mar Drugs. 2019 Jul 11;17(7):410. doi: 10.3390/md17070410 (PMC6669709; doi:10.3390/md17070410)
Supplement: Supplementary file 1 [file marinedrugs-17-00410-s001.pdf]

Supporting Information

Identification and Total Synthesis of Two Previously Unreported Odd-Chain Bis-Methylene-Interrupted Fatty Acids with a Terminal Olefin that Activate Protein Phosphatase, $Mg2^{+}/Mn2^{+}$-Dependent 1A (PPM1A) in Ovaries of the Limpet *Cellana toreuma*

Hideki Kawashima, Naoki Toyooka, Takuya Okada, Huy Du Nguyen, Yuya Nishikawa, Yuka Miura, Nana Inoue, Motoko Ohnishi, and Ken-ichi Kimura

The Table of Contents

Figure S1 ^{1}H and ^{13}C NMR spectra of **4b**······S1

Figure S2 ^{1}H and ^{13}C NMR spectra of **6a**······S2

Figure S3 ^{1}H and ^{13}C NMR spectra of **6b** ······S3

Figure S4 ^{1}H and ^{13}C NMR spectra of synthesized compound **1**······S4

Figure S5 ^{1}H and ^{13}C NMR spectra of synthesized compound **2**······S5

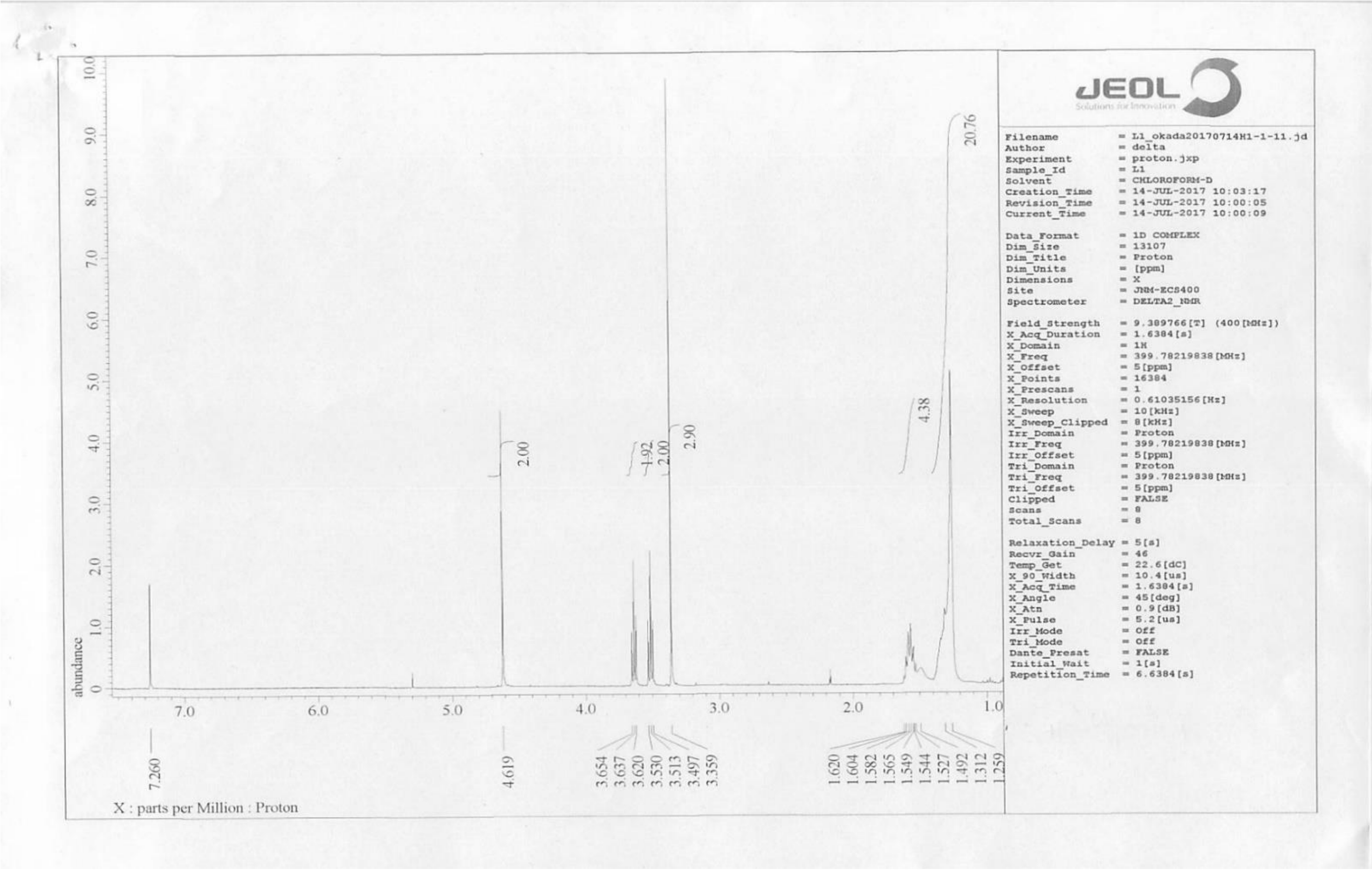

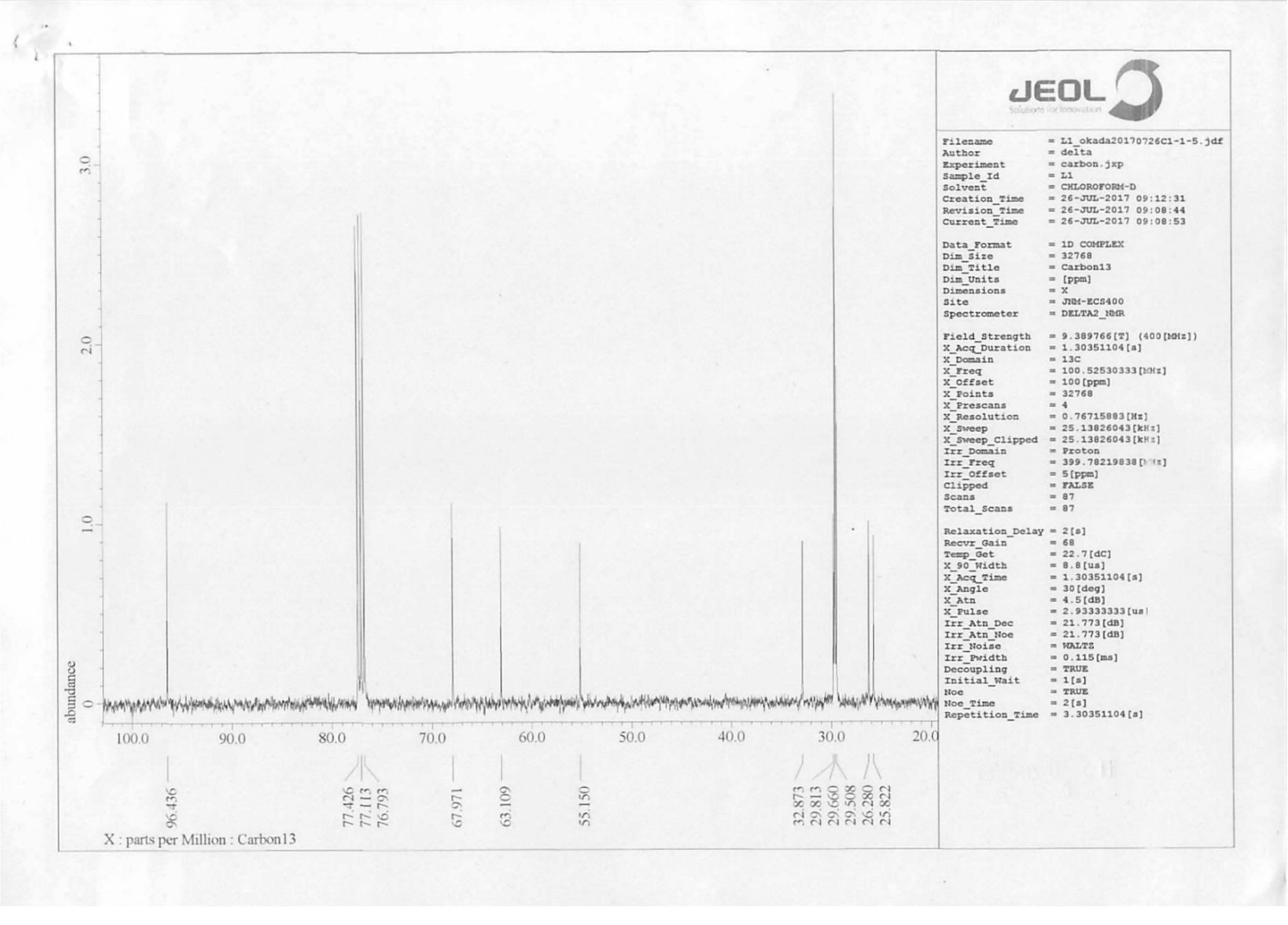

Figure S1 ¹H and ¹³CMR spectra of **4b**

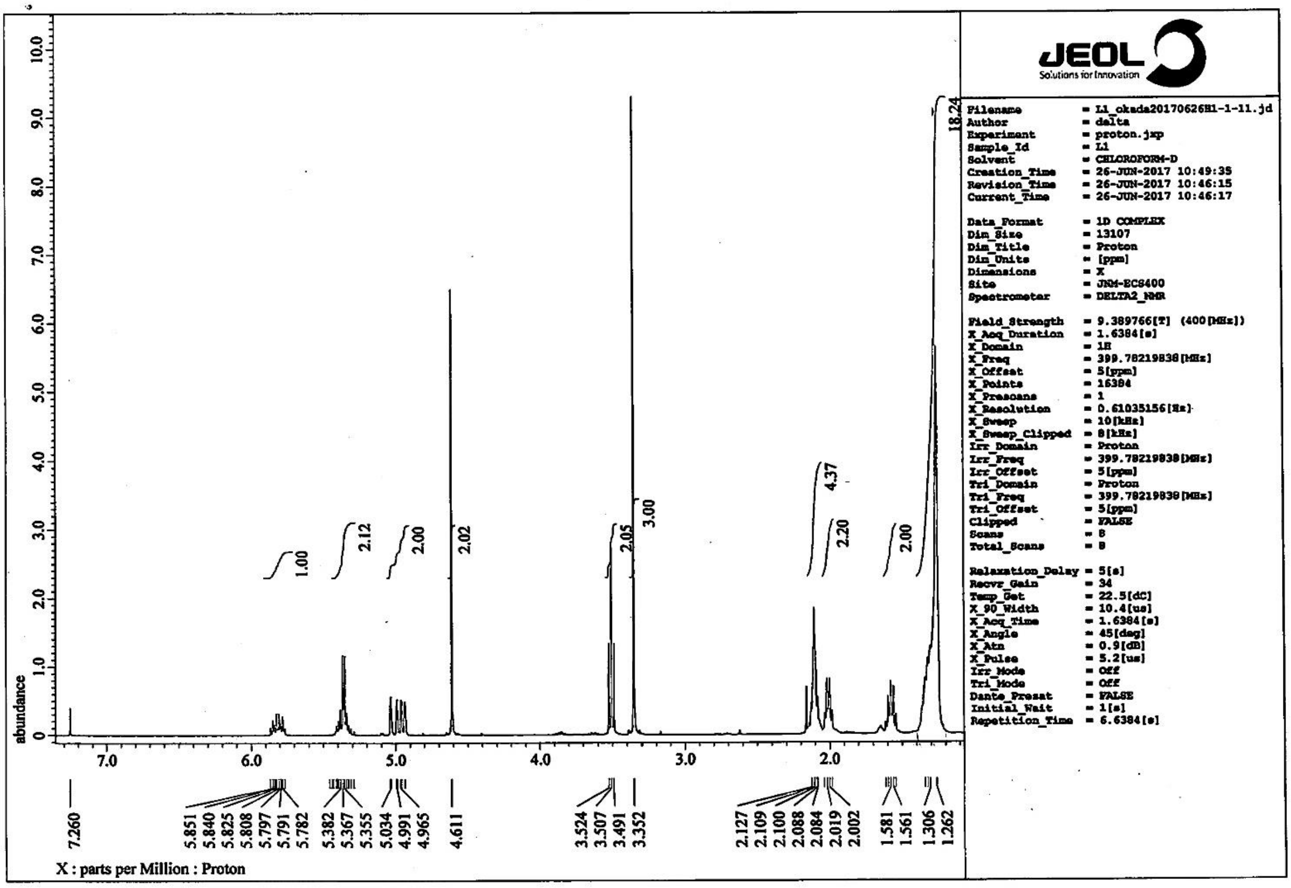

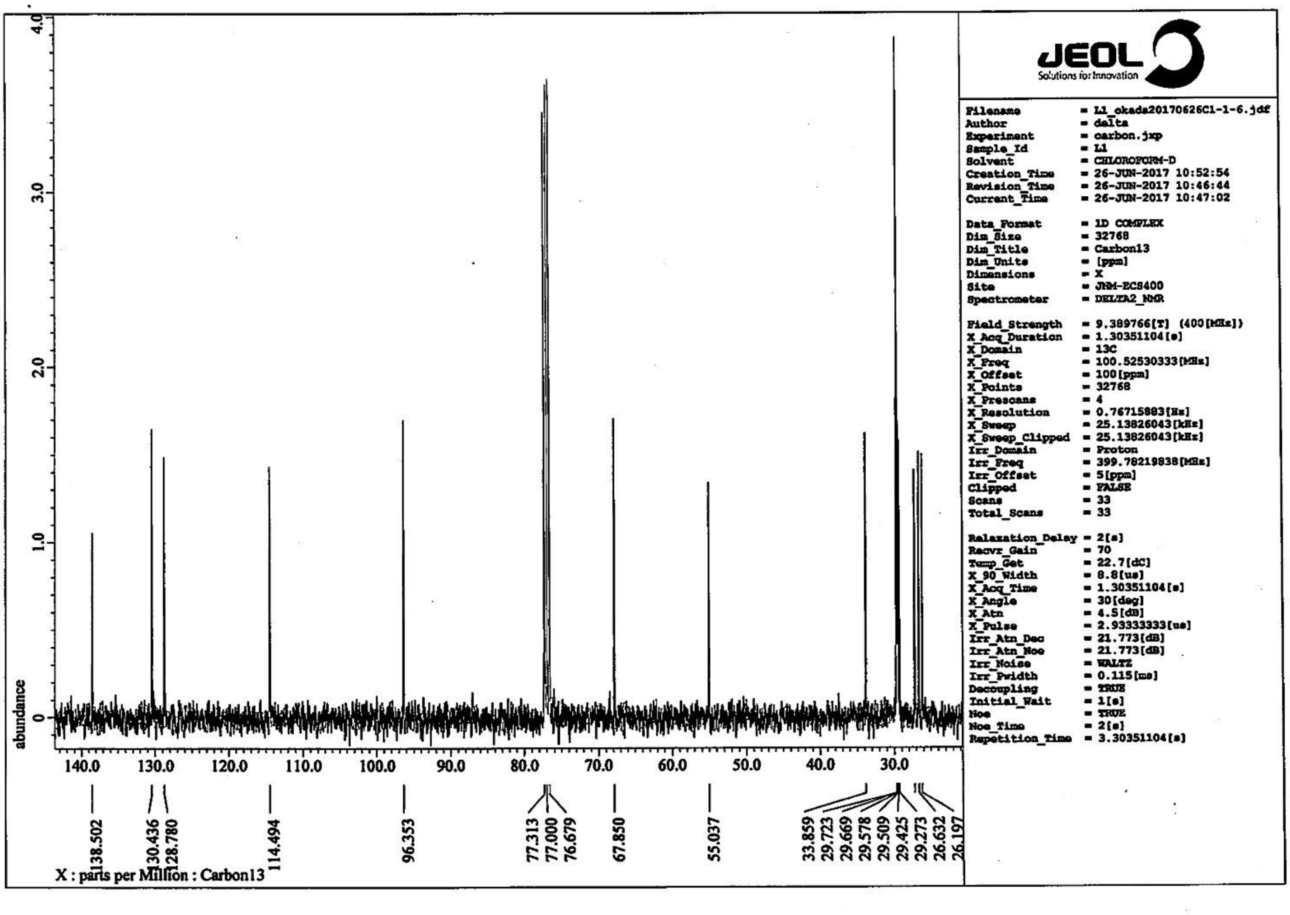

Figure S2 ^{1}H and [13]CMR spectra of **6a**

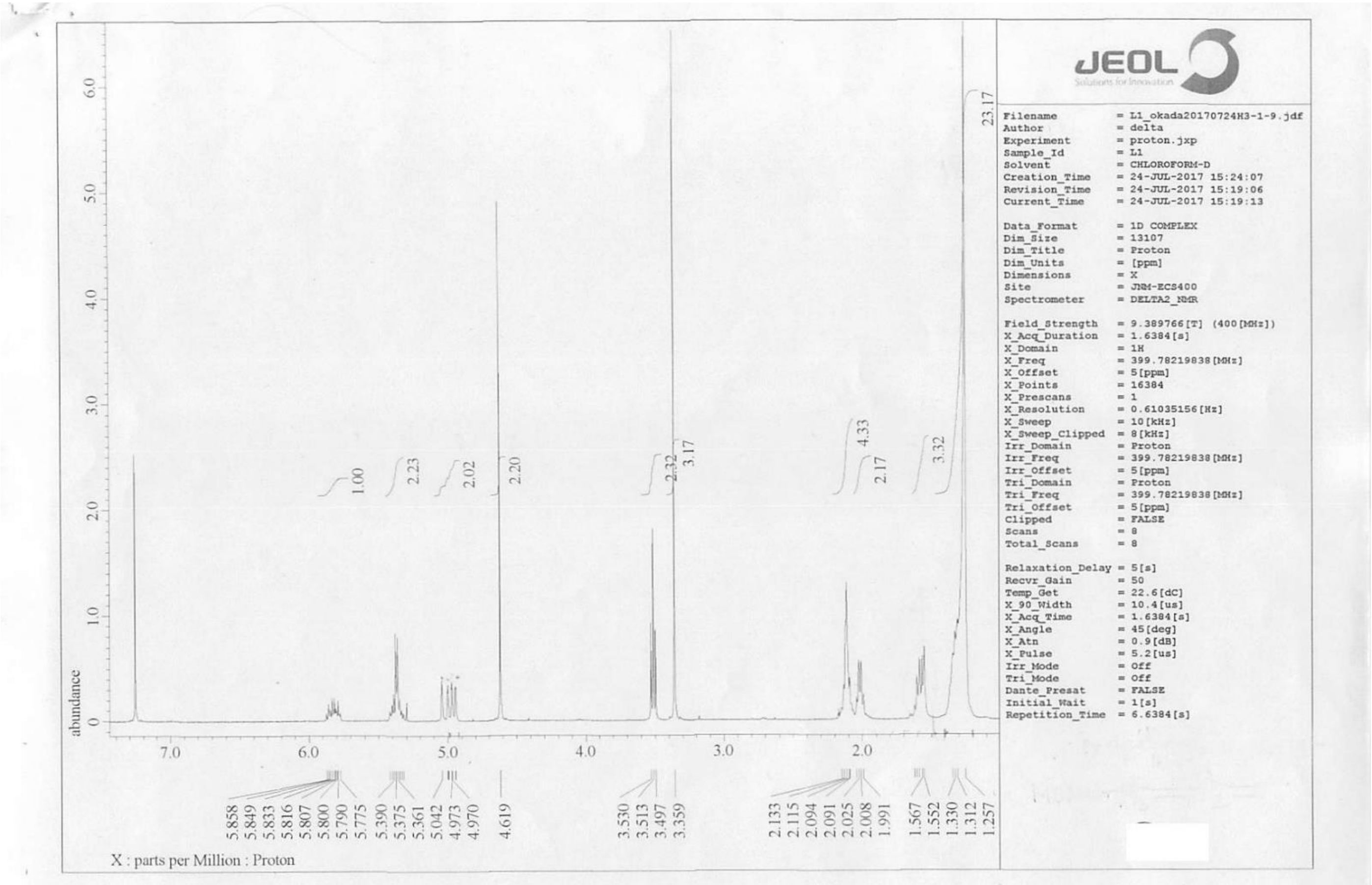

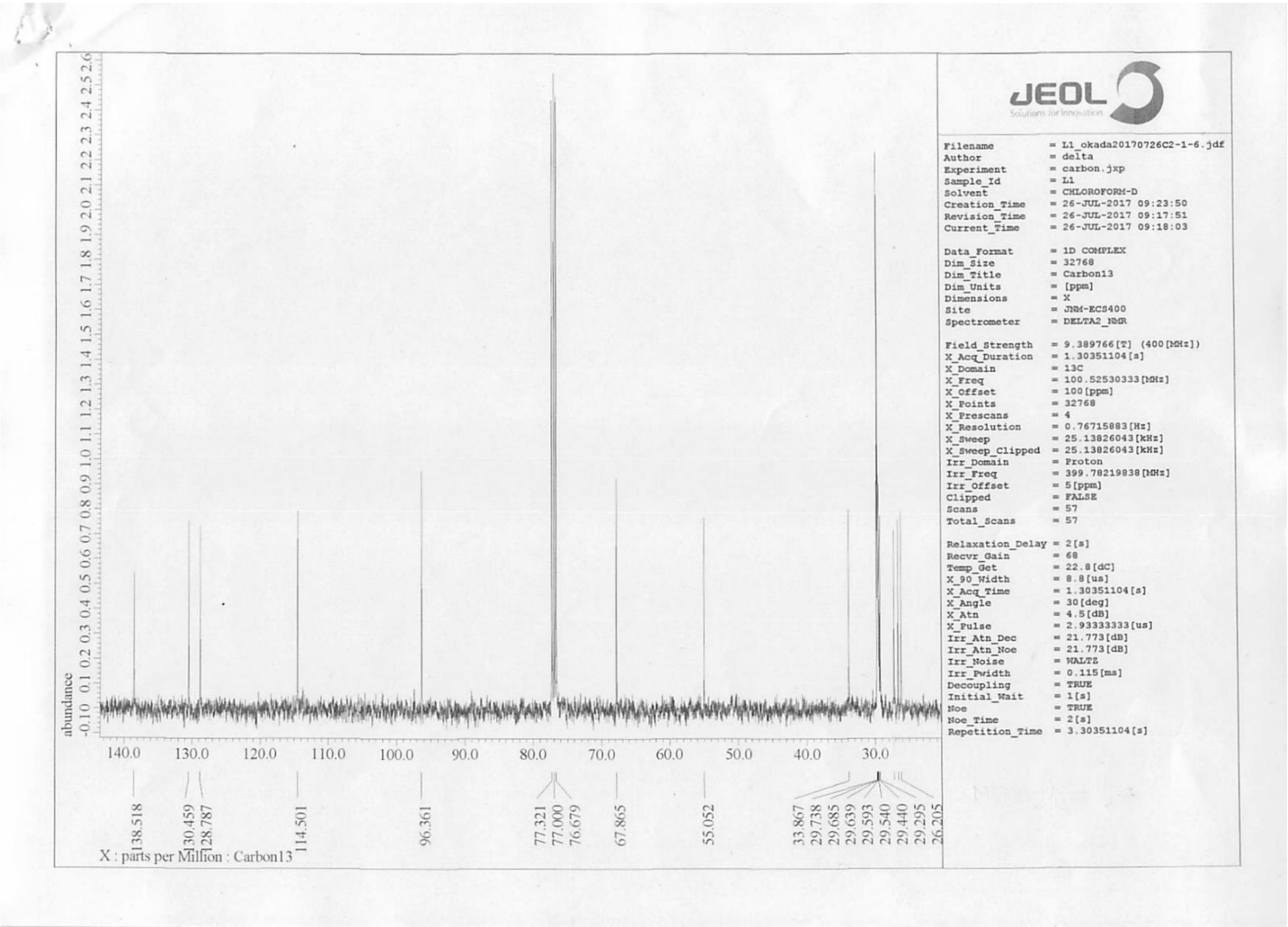

Figure S3 ^{1}H and [13]CMR spectra of **6b**

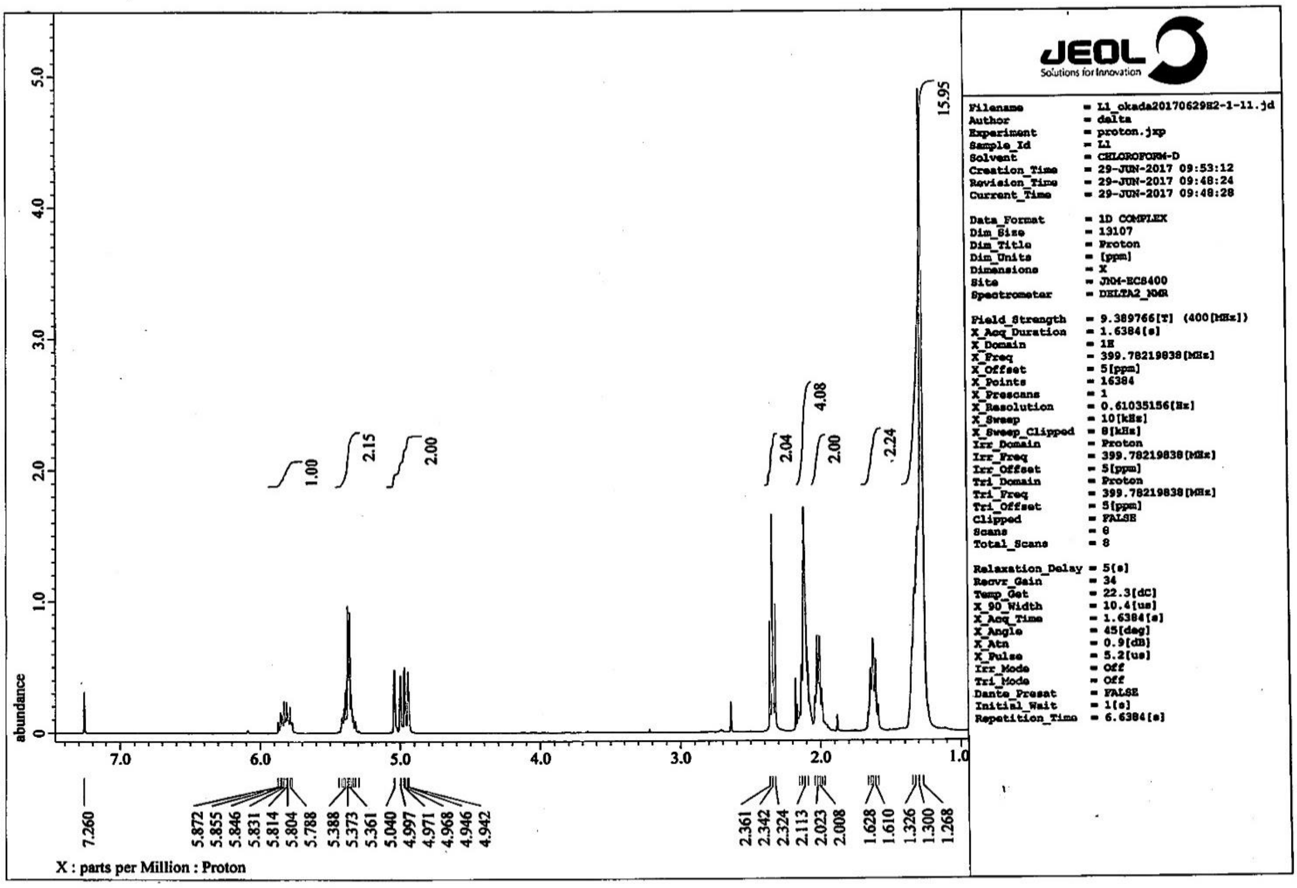

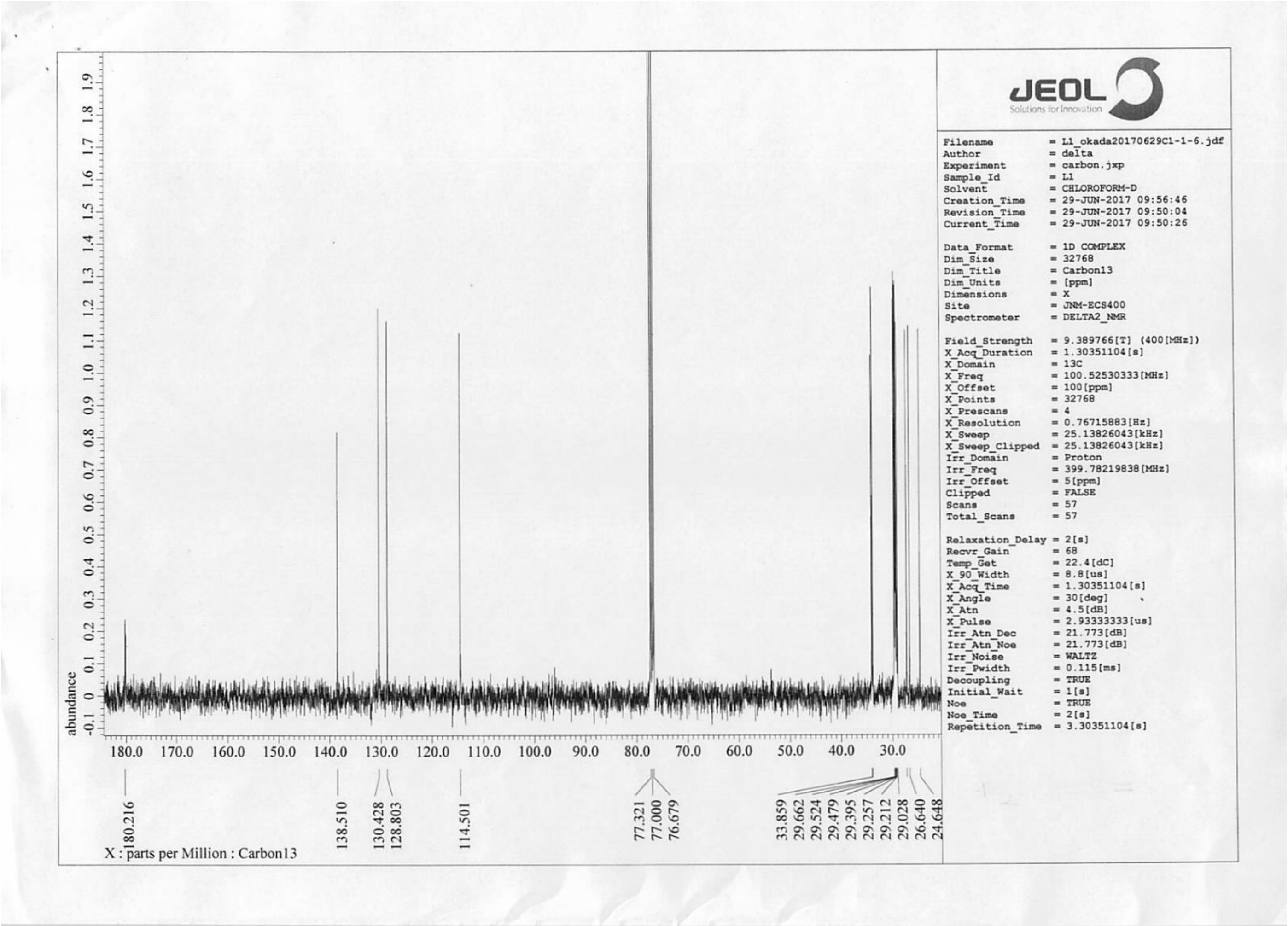

Figure S4 ^{1}H and ^{13}CMR spectra of synthesized compound **1**

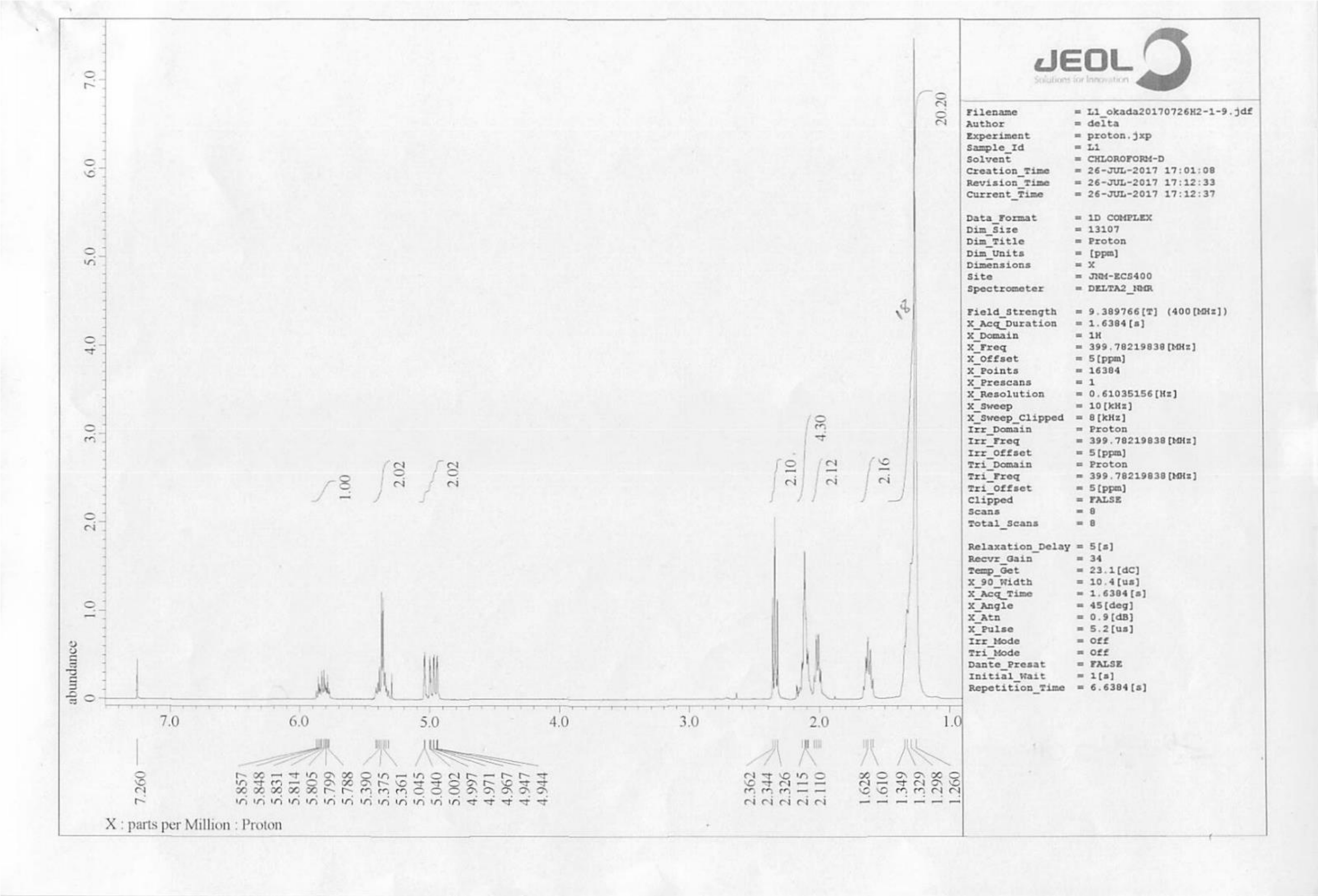

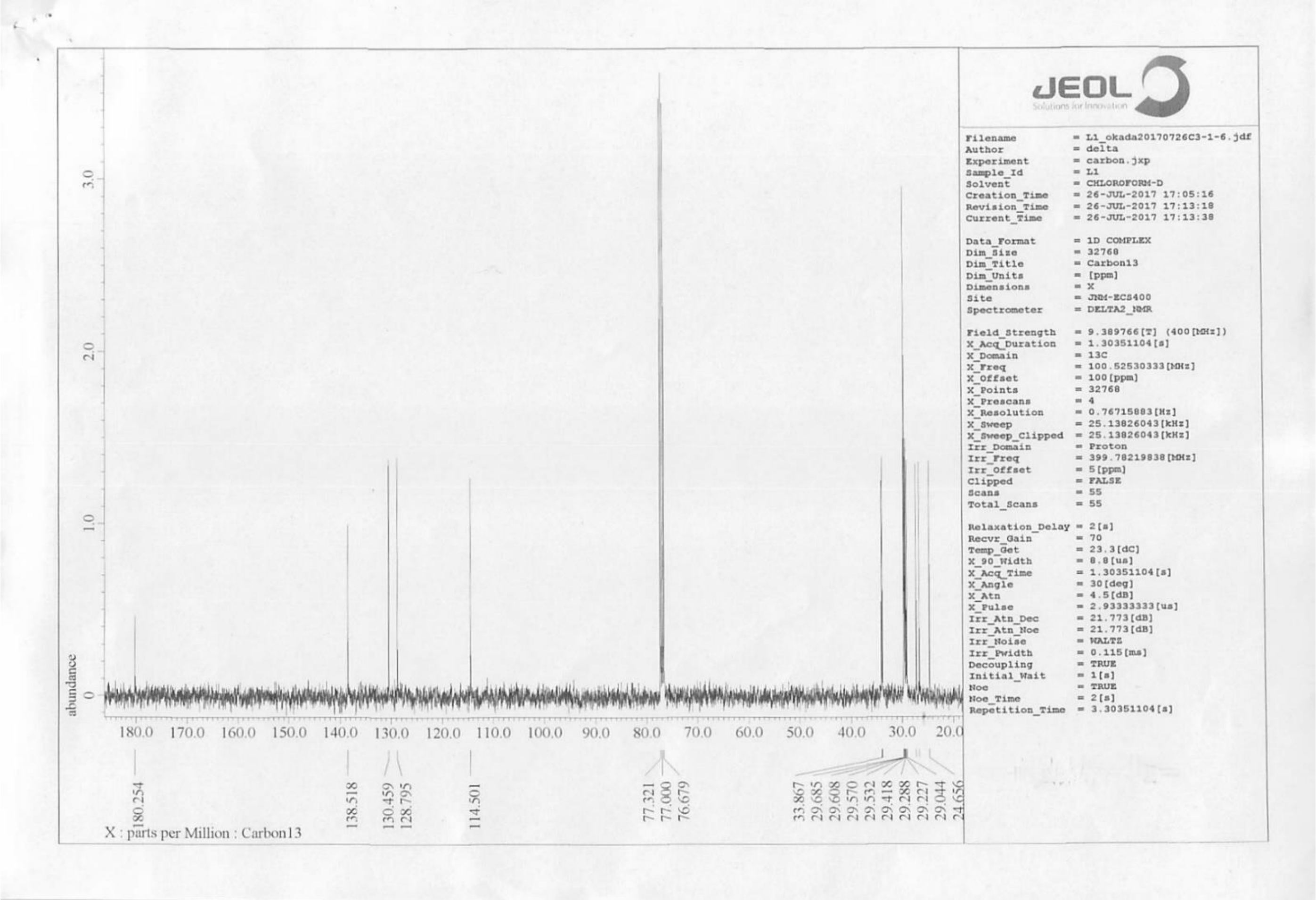

Figure S5 ¹H and ¹³CMR spectra of synthesized compound **2**
